# Human-pathogenic *Anaplasma* spp., and *Rickettsia* spp. in animals in Xi’an, China

**DOI:** 10.1371/journal.pntd.0006916

**Published:** 2018-11-12

**Authors:** Wen-Ping Guo, Baicheng Huang, Qin Zhao, Gang Xu, Baoyuan Liu, Yi-Han Wang, En-Min Zhou

**Affiliations:** 1 Department of Preventive Veterinary Medicine, College of Veterinary Medicine, Northwest A&F University, Yangling, Shaanxi, China; 2 Scientific Observing and Experimental Station of Veterinary Pharmacology and Diagnostic Technology, Ministry of Agriculture, Yangling, Shaanxi, China; Medical College of Wisconsin, UNITED STATES

## Abstract

In China, thirteen species of tick-borne rickettsiales bacteria pathogenic to human have been reported in ticks and host animals, and human patients caused by them also has been identified. However, investigation for rickettsiales bacteria circulating in Xi’an wasn’t performed although diseases resembling human diseases caused by these organisms have been found. In this study, domestic animals and ticks in Xi’an, China, were tested for the presence of rickettsiales bacteria pathogenic to humans. Besides *A*. *ovis*, a high prevalence of *A*. *capra* was observed suggesting a high public health risk exists. In addition, two novel *Anaplasma* species closely related to *A*. *phagocytophilum* were identified and formed distinct lineages in the phylogenetic trees, with more than 98.3% identities for *rrs* gene, while divergences up to 20.2% and 37.0% for *groEL* and *gltA* genes, respectively. Both of these two novel *Anaplasma* species were found to circulate in goats and further assessment of their pathogenicity is needed. *Ca*. R. jingxinensis, with potential pathogenicity, was also detected in *H*. *longicomis* ticks with high prevalence. However, other causative agents were not identified although they were distributed in other areas of China.

## Author summary

China is severely affected by rickettsial diseases caused by rickettsiales bacteria. To date, at least 13 tick-borne rickettsiales bacteria have been reported in China. In this study, we perform molecular epidemiological investigations of rickettsiales bacteria circulating in Xi’an. Through sampling in Xi’an, we show that high prevalence of *Anaplasma capra* in ticks and domestic animals. In addition, *A*. *ovis* and two potentially novel *Anaplasma* species closely related to *A*. *phagocytophilum* are also identified in goats. Besides *Anaplasma*, *Candidatus* Rickettsia jingxinensis, a tentative *Rickettsia* species potential pathogenic to human, is also detected in *H*. *longicomis* ticks with high prevalence. Although our study shows that human-pathogenic rickettsiales bacteria exist in Xi’an, the association between them with the non-viral hemorrhagic fever with renal syndrome (HFRS) cases should be confirmed in further study. In sum, *A*. *capra* and *Ca*. R. jingxinensis infected in domestic animals and ticks with high positive rate, and highlight the high risks for human exposure to *A*. *capra* in Xi’an, China.

## Introduction

Tick-borne rickettsiales bacteria remain an important current and future threat to human and animal health [1, 2]. The members of order Rickettsiales are gram-negative obligate intracellular alphaproteobacteria that can infect numerous eukaryotic hosts [3]. Ticks serve as predominant vectors and hosts for Rickettsiaceae, while only as vectors for Anaplasmataceae [4]. Notably, mammals constitute an important natural host reservoir of Anaplasmataceae through their persistent infections and their key role in supporting survival in the life cycle of these organisms in nature [5].

Of order Rickettsiales, many members are important emerging pathogens, causing anaplasmosis, ehrlichiosis and rickettsiosis in humans or animals [4]. In the last two decades, 13 emerging tick-associated human-pathogenic pathogens have been identified in mainland China, including seven species of spotted fever group *rickettsiae* (SFGR) in Rickettsiaceae; two species of genus *Ehrlichia*, three species of genus *Anaplasma*, and *Candidatus* Neoehrlichia mikurensis in the family Anaplasmataceae [6]. Consistent with the epidemiology of these agents, diseases caused by them have also been reported, such as human granulocytic anaplasmosis (HGA) cases by *A*. *phagocytophilum* [7], human monocytic ehrlichiosis (HME) by *E*. *chaffeensis* [8], rickettsiosis by *R*. *heilongjiangiensis* [9], *Ca*. R. tarasevichiae, *R*. *sibirica*, and *R raoultii*. In addition, patients infected by *A*. *capra*, a novel *Anaplasma* species, were first identified in 2015 [10]; and Rickettsia sp. XY99, a novel spotted fever group Rickettsia, were also first identified in 2016 [11]. However, to date no case caused by other causative agents, including *A*. *ovis*, *A*. *platys*, *E*. *canis*, *R*. *monacensis*, and *R*. *slovaca*, has been yet reported, even though these organisms are prevalent in ticks and animals [6].

Xi’an, the capital city of Shaanxi Province, has been the site of an epidemic of hemorrhagic fever with renal syndrome (HFRS) for many years [12]. Although HFRS is caused by hantaviruses, no viral agent has been detected in a portion of suspected HFRS cases identified by the Xi’an Center for Disease Control and Prevention (CDC). Notably, the clinical symptoms caused by rickettsiales bacteria are easily confused with the clinical presentation of HFRS. However, no molecular epidemiological investigations of rickettsiales circulating in Xi’an have been carried out to determine if these bacteria are circulating in the areas where HFRS cases lacking a confirmed viral etiology have been found. Such studies would be helpful for the both diagnosis of human disease and prevention of tick-borne disease. In this study, we screened domestic animals and ticks in Xi’an for the presence of human pathogens and found that *A*. *capra* and *A*. *ovis* were circulating locally, with *A*. *capra* exhibiting a high infection rate. Also, two potentially novel *Anaplasma* species closely related to *A*. *phagocytophilum* were also detected.

## Materials and methods

### Collection of blood and ticks samples

In 2017, we aseptically collected 281 blood samples from asymptomatic domestic animals frequently contacting with human, including sheep, goats and cattle, in rural villages where non-viral HFRS cases existed from Zhouzhi, Lintong and Chang’an counties in Xi’an city, China. In addition, 24 blood samples from sheep were also from Huyi County where no such case existed. The blood samples were stored into tubes containing EDTA as anticoagulant. The ticks were collected from the animal body surface by hand searching. Ticks were identified by morphology, and then further confirmed by amplifying, sequencing and analyzing their *COI* (cytochrome c oxidase I) gene [13].

### DNA extraction and detection of rickettsiales bacteria

According to the recommended protocol of Tissue DNA Kit (Omega, Norcross, GA, USA), total DNA was extracted from 200μl of whole blood samples. The whole body of each tick was used to extract the total DNA. After extraction, the DNA samples were diluted into 50μl water and stored at -20°C.

DNAs of rickettsiales bacteria were detected by (semi) nest-PCR using primer pairs described previously, targeting *rrs* gene of different taxonomic levels. For family Anaplasmataceae, primer pairs of Ehr1/Ehr2 and Ehr3/Ehr4, for the first and second round of nest-PCR, were used to detect the *Anaplasma*, *Ehrichia* and *Neoehrlichia* [14]. For detection of SFGR, the primer pairs described in our previous study were used [15]. In addition, the specific primers for detection of different species within family Anaplasmataceae were also used to make sure to find out all the positive samples as much as possible [16–24]. The primer sequences were shown in the S1 Table. A DNA sample of *A*. *phagocytophilum* and distilled water were used as positive and negative controls, respectively. In order to avoid contamination, template isolation, PCR preparation, and agarose gel electrophoresis were performed in separate room, and exclusive pipettes and tips were used. The PrimeSTAR GXL Premix including DNA Polymerase with proofreading 3'→5' exonuclease (Takara, Dalian, China) was used in all PCR reaction.

### Amplification of near complete *rrs*, and partial *groEL* and *gltA* genes

In order to obtain the nearly full-length *rrs* gene, primers fD1 and rp2 were used for the first round amplification. In the case of second round amplification, the products of the first round PCR was used as the templates, and primer pairs fD1/Ehr2 and Ehr1/rp2 were used respectively [14, 25].

The *heat shock protein* (*groEL*) and *citrate synthase* (*gltA*) genes were amplified by (semi) nest-PCR using primers described by previous studies [26–28] (S1 Table) and designed by ourselves based on the known sequences of each detected species (see below), and the primer sequences were shown in Table 1 in detail.

**Table 1:** Primer sequences designed in this study.

Pathogens	Target gene	Oligonucleotide sequences (5’- 3’)
*A*. *capra*	*gltA*	ATGATCCGGGGTTCCTGTC (+)
		TGCAGGTCTGAGATAACCT (+)
		TACAATACCGGAGTAAAAGT (-)
	*groEL*	GCGAGGCGTTAGAYAAGTCCAT (+)
		ATGAAGAGCATMAARCCCG (+)
		CYAGAGATGCAAGCGTGTATAG (-)
*A*. *ovis*	*gltA*	GTGAGCTTGCCGACTTTGT (+)
		GTTCTTGTAGACYCTGTGG (-)
		ATGAGTCTCACTCCGCTCT (-)
	*groEL*	GAYGCTGTTGGTTGCACTGC (+)
		ACCGTYGCTATTAGCAAGCC (+)
		AGTGACACAGCCARGTCAAAC (-)
*Anaplasma* sp.	*groEL*	GGWCTKACTGTMGCMATTAGC (+)
		GGATAYCTWTCTCCWTAYTT (+)
		GCTYTWAGCACATTGGTRCT (+)
		TCWGARCYACTGTCRACACT (-)
		ATGCTKGGTGATATAGCDGT (+)
		CGARCTTGCWGTWAAGATGG (+)
		GAACACYGCWGCAAGAGARAC (-)

### Sequencing of the PCR products

PCR products of expected size amplified by each of the primer sets were purified using Gel Extraction kit (TaKaRa, Dalian, China). The purified DNA was ligated into T-vector pMD19 (Takara, Dalian, China). Recombinant plasmids were transformed into *E*. *coli* and positive inserts were confirmed by PCR. For each PCR amplicon, three positive clones were submitted for sequencing of each PCR product to determine the consensus sequence. For PCR products amplified by the primer sets for universal screening family Anaplasmataceae and genus *Rickettsia*, twenty positive clones were sequenced for each PCR product to determine the consensus sequence or the co-infection.

### Sequences analysis

The sequences obtained in this study were edited and assembled using Bioedit v.7.1.11 [29]. Multiple alignments of the nucleotide sequences were performed using the CLUSTAL method in the software MEGA 7.0 [30]. The nucleotide identities were calculated using the Lasergene program [31]. The optimal nucleotide substitution model for different gene data for phylogenetic analysis was determined using software jModelTest [32]. Maximum likelihood (ML) method implemented in PhyML v3.2 was used to reconstruct the phylogenetic tree [33]. Bootstrap analysis with 100 replicates was used to test the reliability of branches in the tree, and the value more than 70% was considered significant and shown. The tree was midpoint-rooted and shown by MEGA 7.0.

### Statistical analysis

SPSS software (Version 20, IBM, Armonk, NY, USA) was used to calculate the *P* value with Chi-square test to determine the significance in different sampling sites and animals hosts. A probability of 0.05 was considered to be threshold.

### Ethics statement

Animal experiments were performed according to *Guidance for Experimental Animal Welfare and Ethical Treatment* by the Ministry of Science and Technology of China (http://www.most.gov.cn/fggw/zfwj/zfwj2006/200609/t20060930_54389.htm). Experimental procedures and animal use and care protocols were carried out in accordance with the guidelines of the Northwest A&F University Institutional Committee for the Care and Use of Laboratory Animals with the license number NWAFU 142 (Shan) 20171124/09 and were approved by the Committee on Ethical Use of Animals of Northwest A&F University.

## Results

### Collection of samples

In 2017, 305 blood samples from asymptomatic sheep, goats, and cattle in rural villages were collected; ticks were also collected from the animals, but due to farmers’ use of pesticides only 202 ticks were collected (Table 2). One tick was collected from each sheep, goat or cattle, and all ticks had sucked blood from the animals. One third of the ticks, including all positive ones for rickettsiales bacteria, as well as the samples that were uncertain by morphological identification, were selected to be identified using molecular biology approaches. The *COI* gene of 171 ticks had approximate 99.4% identities with that of *Rhipicephalus microplus* deposited in GenBank by blast, including 11 nymphal and 160 adult ticks. Hence, these ticks were identified as *R*. *microplus* based on the morphology and analyzing the *COI* gene. In addition, another 31 adult ticks were identified as *Haemaphysalis longicornis*, sharing approximate 99.0% identities with known sequences.

**Table 2:** Prevalence of *A*. *capra*, *A*. *phagocytophilum* and *A*. *ovis* in goat, sheep, cattle and ticks in Xi’an, China.

County	Species	No. tested	No. Positive				
			*A*. *capra*	*A*. *ovis*	*Ca*. A. dongdaense	*Ca*. A. zhouzhiense	*Ca*. R. jingxinensis
Zhouzhi	Goat	36	0	0	0	3	0
	Cattle	12	1	0	0	0	0
	Tick	56	14	0	0	0	0
Huyi	Sheep	24	0	0	0	0	0
Lintong	Sheep	24	0	0	0	0	0
	Goat	23	2	0	2	0	0
	Cattle	12	1	0	0	0	0
	Tick	61	20	0	0	0	0
Chang’an	Sheep	47	10	0	1	0	0
	Goat	115	15	1	8	0	0
	Cattle	12	0	0	0	0	0
	Tick	85	42	0	0	0	14

### Detection of rickettsiales bacteria by PCR

A total of 29 *rrs* gene sequences from 2 cattle, 10 sheep, and 17 goats, amplified with the primers for *A*. *capra*, were consistent with the expected size. Sequencing and further blast showed that all these were identified as *A*. *capra* (Table 2), with an overall prevalence of 9.5%. Infection rates in various sampling sites ranged from 0 to 14.4%; infection rates were 9.8% in goat and 10.5% in sheep, higher than 5.6% in cattle (*P*<0.05). In addition, *A*. *ovis* was discovered in 1 goat. However, no other causative agents pathogenic to human were detected although they had been identified in other parts of China, even the widely distributed *A*. *phagocytophilum*. Although *A*. *phagocytophilum* was not identified in this study, 14 *rrs* gene sequences from *Ca*. A. zhouzhiense and *Ca*. A. dongdaense close related to it were found in goats. Co-infection between *Ca*. A. dongdaense and *A*. *capra* was detected from one goat sample in Chang’an County.

In addition, seventy-six *rrs* gene sequences of *A*. *capra* were also obtained from ticks, showing high infection rates approaching 37.6% and no significant rate difference between nymphal and adult ticks. And positive rates of *A*. *capra* in various sampling sites also different, ranging from 25.0% to 49.4%. For *R*. *microplus*, the positive rate of *A*. *capra* was 40.4%, while 22.6% for *H*. *longicornis*. Significant rate difference was observed between this two tick species (*P*<0.01). Similarly, no other causative agents pathogenic to human within family *Anaplasmataceae* were detected from tick samples. However, a rickettsia species, sharing the closest relationship with *Rickettsia* clone YN03 (KY433580), uncultured *Rickettsia* sp. clone HtFM4 (KU758903), uncultured *Rickettsia* sp. clone HtM69 (KU758904), *Rickettsia* sp. clone SY103 [34], *Rickettsia* sp. strain WHBMXZ-80 and *Rickettsia* sp. strain WHBMXZ-90-1 [35], was only detected from 14 *H*. *longicornis* ticks in Chang’an County, with a 45.2% positive rate. Co-infection between it and *A*. *capra* was found in 5 longhorned ticks.

### Genetic and phylogenetic analysis

In order to better identify and characterize the rickettsiales bacteria, nearly complete *rrs* gene and partial *gltA* and *groEL* gene sequences were also obtained and analyzed from the DNA-positive ticks.

**Fig 1:**
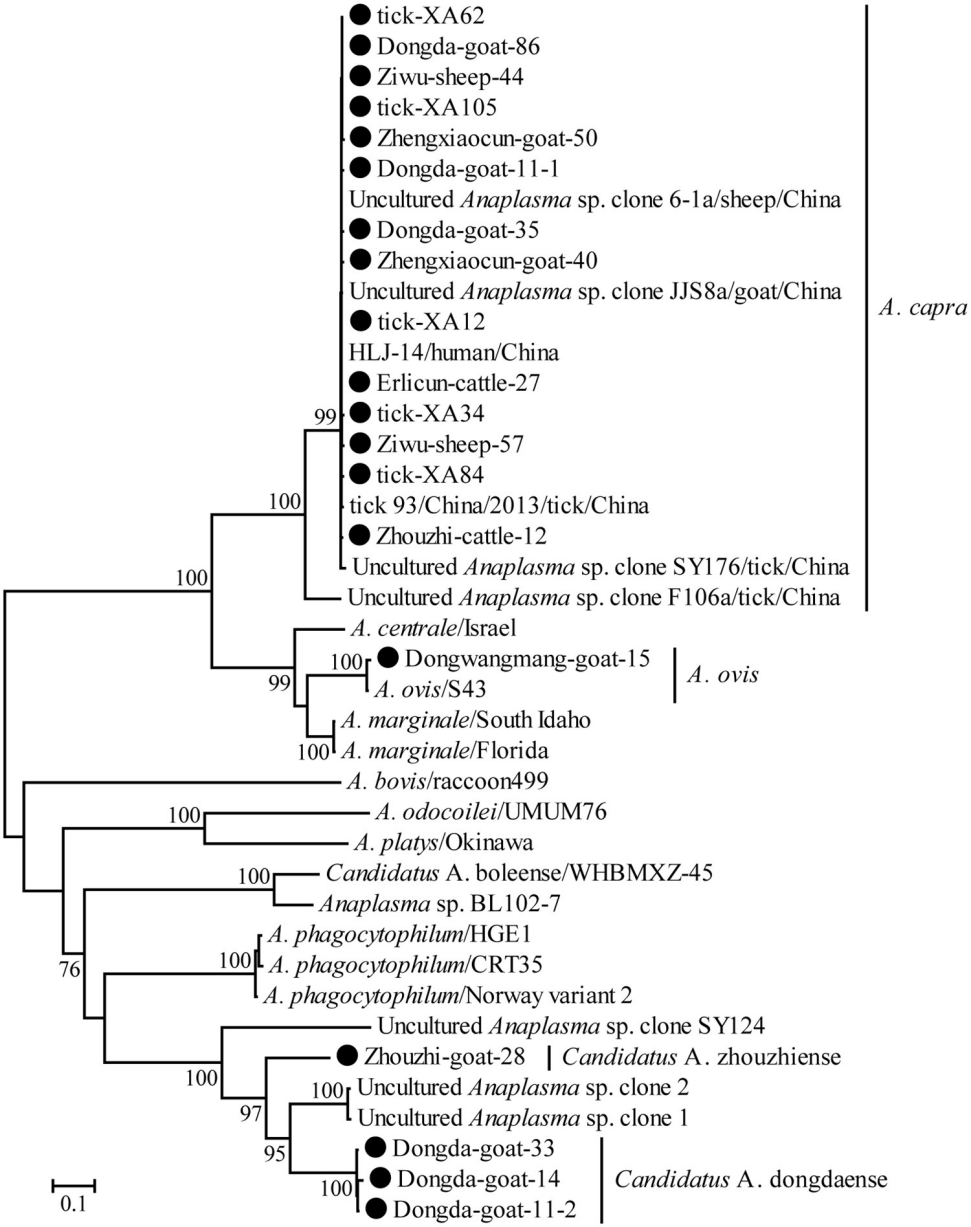
Phylogenetic tree of polymorphisms from *rrs*, *groEL* and *gltA* genes sequences of genus *Anaplasma* to better understand their genetic relationship with the known sequence. Numbers at each node indicate bootstrap values. The tree was mid-point rooted for clarity and the scale bar represents the number of nucleotide substitutions per site. The representative strains obtained in the present study were used to reconstruct the tree and marked by circles.

Almost full-length *rrs* DNA sequences (>1,400 bp) (n = 98), the *gltA* gene (n = 94), and *groEL* gene (n = 90) were obtained from *A*. *capra*-positive samples (n = 98). The sequences were closely related to each other, with 99.4–100% nucleotide identity, and also shared high identity with other *A*. *capra* strains (87.5–100% identity). The tree based on *rrs*, *groEL* and *gltA* gene sequences and the polymorphisms from three genes was reconstructed based on the GTR+Γ+I model. In the phylogenetic tree, *A*. *capra* fell into two clades (Figs 1 and S1). One clade included uncultured *Anaplasma* sp. F106a, *Anaplasma* sp. NS104, *Anaplasma* sp. Kamoshika17 and uncultured *Anaplasma* sp. c1-22b. The other clade was comprised of sequences recovered from this study, human (HLJ-14) in China, and other previously identified strains from goats, sheep, ticks, cattle (defined as *A*. *centrale* previously), deer, and serows in Japan, all which shared close genetic relationship with each other.

Strain Dongwangmang-goat-15 had the highest nucleotide identities with other *A*. *ovis* strains. The *rrs* gene shared 100% identities with strain S43 from China, as well as 1/11/2/480 sheep 3573/7 from South Africa. The *groEL* gene shared 99.7% identities with strain OVI from Xinjiang of China. The *gltA* gene shared 100% identities with strain TC248-1 from Xinjiang of China. Consistent with the nucleotide identities, Dongwangmang-goat-15 clustered together with strains S43, TC249-5 and OVI in all four phylogenetic trees (Figs 1 and S1).

Two *A*. *phagocytophilum*-related species, designed as *Ca*. A. zhouzhiense and *Ca*. A. dongdaense, were found in goats, which exhibited 98.3–98.8%, 79.8–80.7%, and 63.0–64.4% identities with *A*. *phagocytophilum* for *rrs*, *groEL*, and *gltA* genes, respectively. In the *rrs* tree, *Ca*. A. zhouzhiense (n = 3) clustered with uncultured *Anaplasma* sp. JC3-4, while *Ca*. A. dongdaense (n = 11) clustered with both uncultured *Anaplasma* sp. JC3-5 and other strains defined as *A*. *phagocytophilum* (Figs 1 and S1A). *Ca*. A. zhouzhiense formed a separate lineage in the *groEL* tree (S1B Fig), while sharing a cluster with ZJ56 (defined as *A*. *phygocytophilum*) in the *gltA* tree (S1C Fig). *Ca*. A. dongdaense clustered with SHX21 and HN238 (defined as *A*. *phygocytophilum*) in the *groEL* tree (S1B Fig), while forming a separate lineage in the *gltA* tree (S1C Fig).

**Fig 2:**
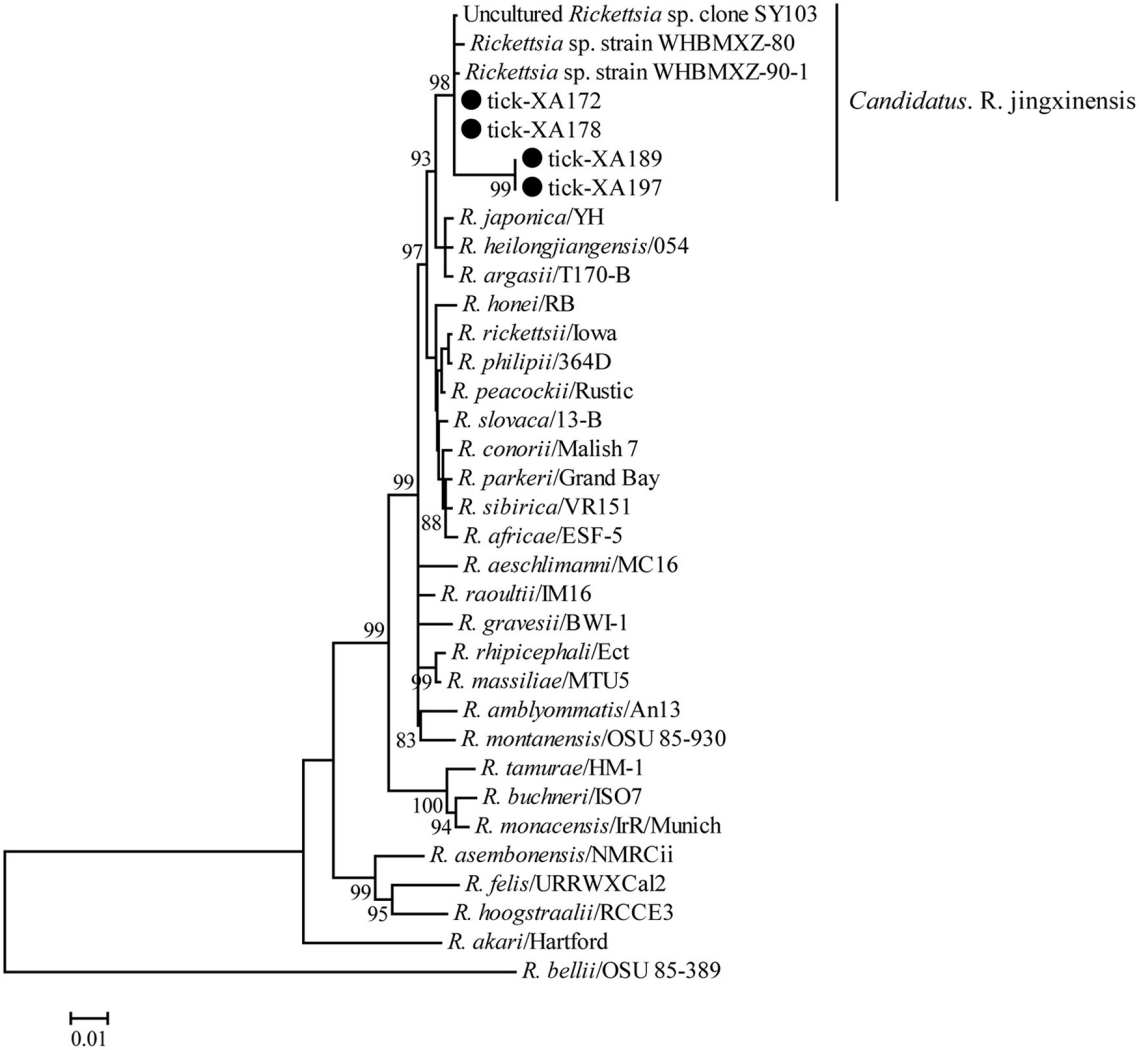
Phylogenetic tree of polymorphisms from *rrs*, *groEL* and *gltA* genes sequences of genus *Rickettsia* to better understand their genetic relationship with the known sequence. Numbers at each node indicate bootstrap values. The tree was mid-point rooted for clarity and the scale bar represents the number of nucleotide substitutions per site. The representative strains obtained in this study were used to reconstruct the tree and marked by circles.

The *Rickettsia* species detected in this study presented 99.5–99.7%, 99.9–100% and 99.8–100% identities with other variants of *Ca*. R. jingxinensis for *rrs*, *groEL*, and *gltA* genes, respectively. In the tree, they also clustered with *Rickettsia* sp. clone SY103 [34], *Rickettsia* clone YN03 (KY433580 and KY433588), uncultured *Rickettsia* sp. clone HtFM4 (KU758903), uncultured *Rickettsia* sp. clone HtM69 (KU758904), *Rickettsia* sp. strain WHBMXZ-80 and *Rickettsia* sp. strain WHBMXZ-90-1 [35], *Ca*. R. jingxinensis isolates Hl13 and Hl14 [36], *Rickettsia* endosymbiont of Haemaphysalis longicornis isolate tick47 (KY617774), *Rickettsia* sp. LON-13 (AB516964), *Rickettsia* sp. Mie180 (JQ697958), and *Rickettsia* sp. isolate XY118 (KU853023) detected from blood sample of human in China (Figs 2 and S2).

## Discussion

To date, this is the first molecular survey of rickettsiales bacteria pathogenic to humans in Xi’an, China. We identified *A*. *capra* and *A*. *ovis* DNA in the blood of sheep, goats and, cattle. In addition, two novel *Anaplasma* species close related to *A*. *phagocytophilum* were identified in goats. The positive rate of *A*. *capra* in animals was 9.5%, in agreement with previous studies; while similar infection rates were observed in sheep and goats, in contrast to the conclusions of a previous study [37]. This discrepancy may be due to contributing factors unique to each of the different sampling sites. In addition, *A*. *capra* was also identified in cattle in China and Japan (previously identified as *A*. *centrale*), suggesting that cattle are also a host reservoir for *A*. *capra*, although it was named based on the host from which it was identified firstly. Another notable thing is that the identification of *A*. *capra* based on *rrs* gene is not enough and it was confused with *A*. *centrale*, and at least one other gene should be considered.

Additionally, two hard ticks were collected, and they were *R*. *microplus* and *H*. *longicornis*, which were important vectors for rickettsiales bacteria pathogenic to humans [35, 38]. In the present study, high prevalence, approaching 37.6%, of *A*. *capra* was found in *R*. *microplus* and *H*. *longicornis* ticks, higher than that from *I*. *persulcatus* (3%) and *H*. *longicornis* (0.43%) in previous studies [10, 39]. Moreover, higher positive rate of *A*. *capra* in *R*. *microplus* than that in *H*. *longicornis* in the present study. Totally, our data showed that *R*. *microplus* was also a vector for *A*. *capra*, with high positive rate in Xi’an, China. It is a remarkable fact that *R*. *microplus* is widely distributed in China and worldwide [40] and habited in the body of livestock close to humans, suggesting it may be a substantial health threat to human beings.

*A*. *capra*, a novel anaplasma species, was found from 28 patients and confirmed to be a pathogenic to humans in 2015. Patients infected by *A*. *capra* mainly presented fever, headache, malaise, dizziness and chills. *A*. *ovis* was detected in China and around the world for many years since its first description [41–43]. In 2010, a variant of *A*. *bovis* was verified to be a potential zoonotic pathogen according to the detection in a human mainly presenting fever [44]. For HFRS, the moderate clinical symptoms or the early stage of severe HFRS also mainly presented fever, headache, malaise, dizziness and chills, the same as the clinical manifestation infected with *A*. *capra* and *A*. *ovis* [45–48]. Therefore, physicians with no experience should be aware of the possibility of *A*. *capra* infection for above-mentioned HFRS cases without a confirmed viral etiology and detected the presence of *A*. *capra* and *A*. *ovis*. The causative agent of these should be confirmed by laboratory diagnosis, and doxycycline should be used to treat these cases if they were infected with *A*. *capra*. However, no human samples were tested to confirm the association between *Anaplasma* and these non-viral HFRS cases although high infection of *A*. *capra* in ticks. Therefore, the actual causative agents for these patients should be identified in further study.

To date, six species have been identified in the genus *Anaplasma* [3, 6, 10]. In addition, an increasing number of sequences that are highly homologous to sequences of the *Anaplasma* genus have been obtained from ticks, animals, and patients [6, 10]. If fact, in this study, both *Ca*. A. zhouzhiense and *Ca*. A. dongdaense were detected. Although their sequences clustered most closely with several strains classified as *A*. *phagocytophilum*, these organisms may represent novel species within the *Anaplasma* genus. This possibility is supported after comparison of their sequences with those of known species, as well as their positions in the phylogeny. Moreover, further studies are required to determine the pathogenicity of these organisms toward humans. Similar with the identification of *A*. *capra* based on *rrs* gene, the nucleotide sequences obtained from the two primer sets, one for detection of Anaplasmataceae and the other for *A*. *phagocytophilum*, shared more than 99.0% identity, and these two species may be wrongly identified as *A*. *phagocytophilum*. Hence, the full-length *rrs* gene or other genes, as the target gene, should be used to identify them rather than partial *rrs* gene.

Similarly, no pathogenic *Rickettsia* species were found in present study although several species and their associated patients had been reported in other parts of China, such as *R*. *heilongjiangiensis*, *Ca*. *R*. *tarasevichiae*, *R*. *sibirica* and *R*. *raoultii* [6]. *Ca*. R. jingxinensis, a novel *Rickettsia* species, was reported in recent years in Shenyang, Wuhan and identified in *R*. *microplus* and *H*. *longicomis* ticks [34–36]. In this study, this species was also identified in *H*. *longicomis* ticks sampled from Xi’an. Hence, these data indicate that this rickettsial agents belonging to spotted fever group (SPG) is co-circulating in various ticks in China broadly. Even more, a variant belonging to *Ca*. R. jingxinensis was identified in human. Therefore, we should pay more attentions to it although its pathogenicity was not confirmed now.

In China, HGA caused by *A*. *phagocytophilum* and HME by *E*. *chaffeensis* have been reported in last two decades; especially *A*. *phagocytophilum* was widely distributed in mammals and ticks [6–8]. Even more, cases of HGA caused by nosocomial transmission were also identified in Anhui Province in 2006 [7]. Although we did not found pathogenic *A*. *phagocytophilum*, *E*. *chaffeensis* and Rickettsia species in Xi’an, this did not mean the no exist of them in the local area. Hence, more investigation should be performed to determine their circulation.

In summary, a survey of *Anaplasma* agents carried by domestic animals and ticks in Xi’an revealed a high prevalence of *A*. *capra* in these populations. People living in rural areas, who tend to have a higher likelihood of coming into contact with ticks and domestic animals, are more likely to be infected. Indeed, as shown in this study, people living in Xi’an are at high risk of exposure to *A*. *capra* and *A*. *capra* could be considered a public health threat in Xi’an, China. Further epidemiological studies are needed to assess the scope of this threat to human health. In addition, the pathogenicity of two novel *Anaplasma* species characterized in this study and *Ca*. R. jingxinensis should be further assessed. Simultaneously, specific diagnostic kits should be developed to test these rickettsiales bacteria pathogenic to human.

## Supporting information

S1 Fig Phylogenetic tree of *rrs* (A), *groEL* (B) and *gltA* (C) gene sequences of genus *Anaplasma*. Numbers at each node indicate bootstrap values. The tree was mid-point rooted for clarity and the scale bar represents the number of nucleotide substitutions per site. The representative strains obtained in this study were used to reconstruct the tree and marked by circles.
(TIF)

S2 Fig Phylogenetic tree of *rrs* (A), *groEL* (B) and *gltA* (C) gene sequences of genus *Rickettsia*. Numbers at each node indicate bootstrap values. The tree was mid-point rooted for clarity and the scale bar represents the number of nucleotide substitutions per site. The representative strains obtained in this study were used to reconstruct the tree and marked by circles. The *Ca*. R. jingxinensis was shown in bold.
(TIF)

S1 Table Primer sequences used in this study. (DOCX)
