# Supplementary figures and images for: Human-pathogenic Anaplasma spp., and Rickettsia spp. in animals in Xi’an, China
Source: PLoS Negl Trop Dis. 2018 Nov 12;12(11):e0006916. doi: 10.1371/journal.pntd.0006916 (PMC6258427; doi:10.1371/journal.pntd.0006916)

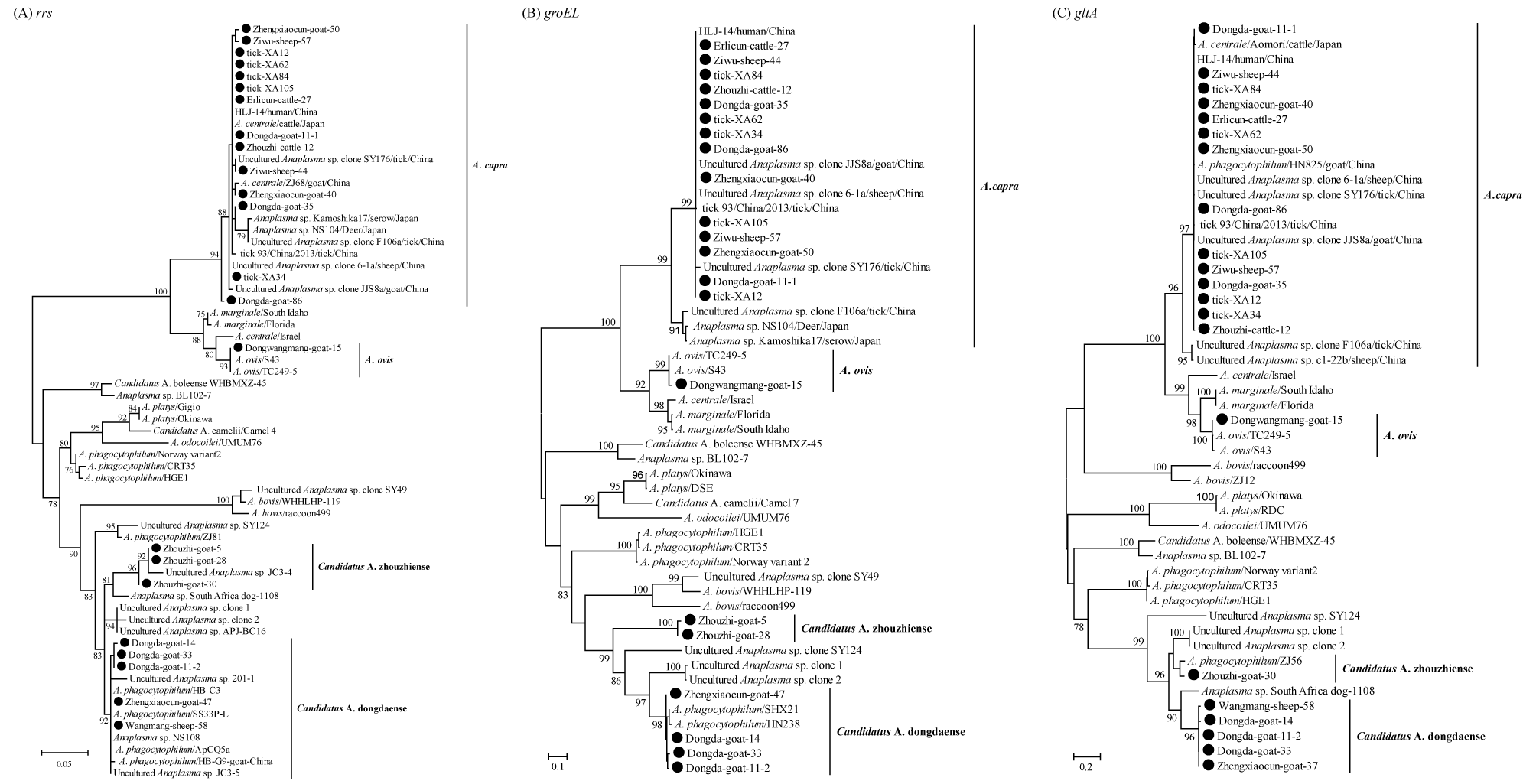

Supplement: S1 Fig — Numbers at each node indicate bootstrap values. The tree was mid-point rooted for clarity and the scale bar represents the number of nucleotide substitutions per site. The representative strains obtained in this study were used to reconstruct the tree and marked by circles. (TIF) [file pntd.0006916.s001.tif]

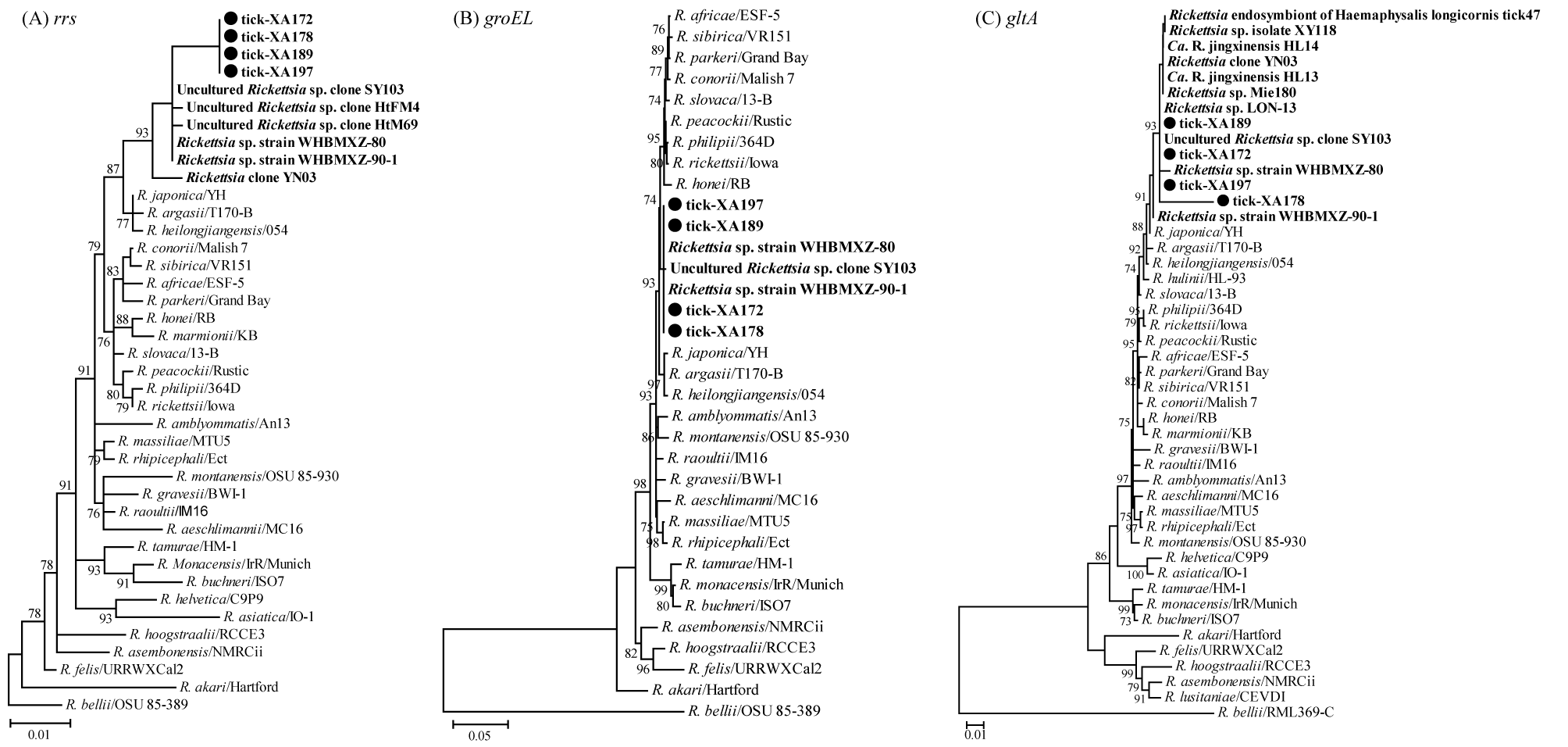

Supplement: S2 Fig — Numbers at each node indicate bootstrap values. The tree was mid-point rooted for clarity and the scale bar represents the number of nucleotide substitutions per site. The representative strains obtained in this study were used to reconstruct the tree and marked by circles. The Ca. R. jingxinensis was shown in bold. (TIF) [file pntd.0006916.s002.tif]
