# Supplementary material for: Human-pathogenic Anaplasma spp., and Rickettsia spp. in animals in Xi’an, China
Source: PLoS Negl Trop Dis. 2018 Nov 12;12(11):e0006916. doi: 10.1371/journal.pntd.0006916 (PMC6258427; doi:10.1371/journal.pntd.0006916)
Supplement: S1 Table — (DOCX) [file pntd.0006916.s003.docx]

| Pathogens | Target gene | Primer | Oligonucleotide sequences (5’- 3’) | References |
| --- | --- | --- | --- | --- |
| Anaplasmataceae | *rrs* | Ehr1 | AACGAACGCTGGCGGCAAGC (+) | 14 |
|  |  | Ehr2 | AGTAYCGRACCAGATAGCCGC (-) | 14 |
|  |  | Ehr3 | TGCATAGGAATCTACCTAGTAG (+) | 14 |
|  |  | Ehr4 | CTAGGAATTCCGCTATCCTCT (-) | 14 |
| *A. phagocytophilum* | *rrs* | EE1 | TCCTGGCTCAGAACGAACGCTGGCG (+) | 17 |
|  |  | EE2 | AGTCACTGACCCAACCTTAAATGGCTG (-) | 17 |
|  |  | SSAP2f | GCTGAATGTGGGGATAATTTAT (+) | 20 |
|  |  | SSAP2r | ATGGCTGCTTCCTTTCGGTTA (+) | 20 |
| *A. capra* | *rrs* | fD1 | AGAGTTTGATCCTGGCTCAG (+) | 25 |
|  |  | rp2 | ACGGCTACCTTGTTACGACTT (-) | 25 |
|  |  |  | GCAAGTCGAACGGACCAAATCTGT (+) | 24 |
|  |  |  | CCACGATTACTAGCGATTCCGACTTC (-) | 24 |
| *A. ovis* | *rrs* | EE1 | TCCTGGCTCAGAACGAACGCTGGCG (+) | 17 |
|  |  | EE2 | AGTCACTGACCCAACCTTAAATGGCTG (-) | 17 |
|  |  | 297E | ACACGGTCCAGACTCCTACG (+) | 23 |
|  |  | 1144R | CTTGACATCATCCCCACCTT (-) | 23 |
| *A. platys* | *rrs* | 8F | AGTTTGATCATGGCTCAG (+) | 221 |
|  |  | 1448R | CCATGGCGTGACGGGCAGTGTG (-) | 21 |
|  |  | PLATYS | GATTTTTGTCGTAGCTTGCTATG (+) | 21 |
|  |  | EHR16SR | TAGCACTCATCGTTTACAGC (-) | 19 |
| *E. chaffeensis* | *rrs* | ECB | CGTATTACCGCGGCTGCTGGCA (+) | 18 |
|  |  | ECC | AGAACGAACGCTGGCGGCAAGCC (-) | 18 |
|  |  | HE1 | CAATTGCTTATAACCTTTTGGTTATAAAT (+) | 16 |
|  |  | HE3 | TATAGGTACCGTCATTATCTTCCCTAT (-) | 16 |

S1Table. Primer sequences used in this study

| Pathogens | Target gene | Primer | Oligonucleotide sequences (5’- 3’) | References |
| --- | --- | --- | --- | --- |
| *E. canis* | *rrs* | ECB | CGTATTACCGCGGCTGCTGGCA (+) | 18 |
|  |  | ECC | AGAACGAACGCTGGCGGCAAGCC (-) | 18 |
|  |  | ECAN5 | CAATTATTTATAGCCTCTGGCTATAGGA (+) | 22 |
|  |  | HE3 | TATAGGTACCGTCATTATCTTCCCTAT (-) | 16 |
| *E. ewingii* | *rrs* | ECB | CGTATTACCGCGGCTGCTGGCA (+) | 18 |
|  |  | ECC | AGAACGAACGCTGGCGGCAAGCC (-) | 18 |
|  |  | EE52 | CGAACAATTCCTAAATAGTCTCTGAC (+) | 22 |
|  |  | HE3 | TATAGGTACCGTCATTATCTTCCCTAT (-) | 16 |
| *Ca. N. mikurensis* | *rrs* |  | GCGACTATCTGGCTCAG (+) | 15 |
|  |  |  | CTATCTGGCTCAGTTCTGAC (+) | 15 |
|  |  |  | TGCCTCCTTACGGTTAG (-) | 15 |
| *Rickettsia* | *rrs* |  | GAAGGCGRTCATYTRGGCT (+) | 15 |
|  |  |  | GRTCATYTRGGCTRCAACTG (+) | 15 |
|  |  |  | CTGCCTCTTGCGTTAGCT (-) | 15 |
| *Anaplasma* sp. | *gltA* | CS7F2 | ATGRTAGAAAAWGCTGTTTT (+) | 27 |
|  |  | HG1085R | ACTATACCKGAGTAAAAGTC (-) | 27 |
|  |  | F1b | GAYCAYGARCARAATGCYTC (+) | 27 |
|  |  | AnaCS1076R | GAGTAAAAGTCGACRTTKGG (-) | 27 |
|  | *gltA* | AP1SPglF1 | ATGBTAGAAAARGCTGTTTTRGMGTGT (+) | 28 |
|  |  | AP1168SPglR1 | TCATACCATTGMGATRCCCATCC (-) | 28 |
|  |  | APJ10F2 | AAKGCTGTTTTAGCGTGTGGTGATCTT (+) | 28 |
|  |  | APJ932R2 | ATTTTCGCCCTCGGGTCGTGA (-) | 28 |
|  | *groEL* | EEgro1F | GAGAGATGCTTATGGTAAGAC (+) | 26 |

(*Continued*)
